# Establishment of a novel assessment of the quality of human spermatozoa measuring mitochondrial oxygen metabolism

**DOI:** 10.1186/s13104-022-06012-4

**Published:** 2022-03-29

**Authors:** Hisanori Taniguchi, Yoshiyuki Matsuo, Kayo Shimoi, Masahiro Yoshimura, Kiichi Hirota, Hidefumi Kinoshita

**Affiliations:** 1 Department of Urology and Andrology, Kansai Medical University, 2-5-1 Shin-machi, Hirakata, Osaka 573-1010 Japan; 2 Reproduction Center, Kansai Medical University Hospital, Hirakata, Osaka Japan; 3 Department of Human Stress Response Science, Institute of Biomedical Science, Kansai Medical University, 2-5-1 Shinmachi, Hirakata, Osaka 573-1010 Japan

**Keywords:** Extracellular flux analysis, Human spermatozoa, Male infertility, Mitochondrial function, Sperm

## Abstract

**Objective:**

We aimed to establish a novel sperm quality evaluation technology by measuring mitochondrial oxygen metabolism in human spermatozoa.

**Results:**

Normozoospermic human sperm samples were used. After establishing the optimal parameters for measuring the oxygen metabolism of human sperm cells using the extracellular flux analyzer, we measured the oxygen consumption rate (OCR) of human spermatozoa exposed to different storage temperatures. Although sperm motility was significantly lower at 4 °C when compared with sperm motility at 37 °C, there were no significant differences in sperm vitality and the OCR under both conditions. The present study established a methodology for human sperm quality evaluation using extracellular flux analysis technology. The OCR evaluation could be a reliable measurement tool for assessing human sperm quality.

**Supplementary Information:**

The online version contains supplementary material available at 10.1186/s13104-022-06012-4.

## Introduction

In treating infertility, clinical assessment of sperm quality is an important issue for determining the appropriate treatment strategy. Semen quality is assessed conventionally by volume, total sperm number, sperm concentration, sperm motility and vitality [1]. Sperm motility is directly dependent on the available energy obtained through ATP hydrolysis, which is produced by mitochondria located in the sperm mid connecting piece [2]. Tourmente et al. used a mouse model to show that sperm with higher oxygen consumption/lactate excretion rate ratios were able to produce greater amounts of ATP and therefore achieve higher swimming velocities [3]. In their study, the mitochondrial metabolism was measured using the Extracellular Flux Analyzer™. The extracellular flux analysis assesses mitochondrial respiration and glycolysis, as well as the ATP production rate of live cells [4, 5]. In this study, we established a reliable method for sperm quality evaluation by measuring oxygen metabolism in sperm mitochondria, and determined the optimal semen sample collection conditions by assessing the effects of storage temperature and duration on energy metabolism, sperm motility and vitality of human sperm.

## Main text

### Materials and methods

#### Study subject

Normozoospermic human sperms from healthy volunteers were used. Three independent semen samples collected before evaluating oxygen metabolism gave normal semen parameter ranges, including semen volume ≥ 1.4 mL, sperm concentration ≥ 16 million/mL and total motility ≥ 42% (standard values according to the World Health Organization (WHO) laboratory manual) [1]. The volunteer had no medical history that indicated possible infertility, such as diabetes mellitus, sexually transmitted diseases, ejaculatory disorders, medically treated psychological illnesses or genetic diseases.

#### Human semen sample preparation

All semen samples were obtained by masturbation into a wide-mouth sterile plastic container in an isolated room just prior to conducting the experiments. All samples were analyzed within 1 h of collection. Sexual abstinence for 3 days was requested. Samples were diluted according to the instructions of the WHO laboratory manual [1]. Sperm concentration, motility and sperm morphology were analyzed by a Sperm Motility Analysis System (SMAS; version 1.0, Kaga Electronics, Tokyo, Japan) [6]. The percentage of motile spermatozoa was classified as progressively motile (WHO class A + B), non-progressively motile (WHO class C) and immotile (WHO class D). Motile spermatozoa (WHO class A + B) were separated by a density gradient system. A fresh semen sample was overlaid on 6.0 mL layers of the ISolate stock solution (Irvine Scientific, Santa Ana, CA, USA) in a conical centrifuge tube. Stirred the boundaries between the semen sample and ISolate stock solution using a pipette. The sample tube was then centrifuged at a low speed of 400×*g* for 30 min. in room temperature. Highly mobile and morphologically normal cells that formed a pellet at the bottom of the sample tube were isolated by an additional centrifugation step at a low speed of 200×*g* for 10 min using the 1000 µL of Universal IVF Medium (ORIGIO Japan, Yokohama, Japan). After isolating highly active, normal sperm from lower quality sperm and other elements, these normal sperm were divided into two samples and incubated at 4 or 37 °C for 2 h.

#### Measurement of sperm vitality

After incubation under the two different conditions, sperm motility and vitality were assessed prior to extracellular flux analysis of oxygen consumption. Sperm motility was assessed by SMAS and vitality was evaluated by eosin-nigrosin staining. Ten microliters of each sample was mixed with staining solution and the sample placed onto a glass slide. Two hundred spermatozoa were examined using a phase contrasted microscope (Olympus, CKX41). Unstained spermatozoa were counted as live cells, and the ratio of living spermatozoa was determined [7].

#### Extracellular flux analysis of human spermatozoa

Reagents used in this study are listed in Additional file 1: Table S1. The XFp cell culture miniplates (Agilent Technologies, Santa Clara, CA, USA) were coated with 0.25 mg/mL concanavalin A (Sigma, St. Louis, MO, USA; 25 µL per well) for 30 min at room temperature, washed thrice with water, and dried for 1 h. Motile spermatozoa were suspended in modified Tyrode’s (mT) solution (131.89 mM NaCl, 2.68 mM KCl, 0.49 mM MgCl, 0.36 mM NaHPO, 1.8 mM CaCl) [3, 8] supplemented with 2 mg/mL bovine serum albumin (BSA), 5 mM glucose, 1 mM sodium pyruvate, and 2 mM glutamine. Cell suspensions prepared in XF Dulbecco’s Modified Eagle’s Medium (XF DMEM, Agilent Technologies) were used for comparison. Sperm cells were seeded onto the coated culture plates at a density of 1 × 10^6^ sperm/well. The plates were then centrifuged at 300*g* for 1 min and cell adhesion to the bottom of the well was confirmed using a microscope (Olympus, CKX41).

The XF Cell Mito Stress Test was carried out according to the manufacturer’s instructions. Briefly, the sensor cartridge for the flux analyzer was hydrated at 37 °C in a non-CO_2_ incubator one day before the experiment. The injection port A on the sensor cartridge was loaded with oligomycin (a complex V inhibitor, final concentration 1 μM), FCCP was loaded into port B, and rotenone/antimycin A (inhibitors of complexes I and III, final concentration 0.5 μM each) were loaded into port C. During sensor calibration, cells were incubated at 37 °C in 180 μl of assay medium (XF DMEM or mT solution) in the non-CO_2_ incubator [9]. The oxygen consumption rate (OCR) and extracellular acidification rate (ECAR) of human sperm were measured using the XFp Extracellular Flux Analyzer™ (Agilent Technologies) with 1 min-mix and 2 min-measure cycles. Three measurements were recorded for each step. (Additional file 2: Figure S1)

The OCR under the two conditions of sperm storage was determined to assess differences in human sperm mitochondrial energy metabolism. The collected same semen sample was divided into two groups and incubated for 2 h at either 4 °C or 37 °C before extracellular flux analysis. A flow chart of the extracellular flux analysis of human spermatozoa is presented in Additional file 3: Figure S2.

#### Statistical analysis

The experiment was repeated three times in triplicate. Data are expressed as the mean ± standard error (SE) and analyzed using the two ways analysis of variance (ANOVA). A *p* value of < 0.05 was considered to be statistically significant. Statistical analyses were performed with SPSS Statistics version 21.0 (IBM Corp., Armonk, NY, USA).

## Results

### Establishing a method for measuring oxygen consumption by human spermatozoa

**Fig. 1:**
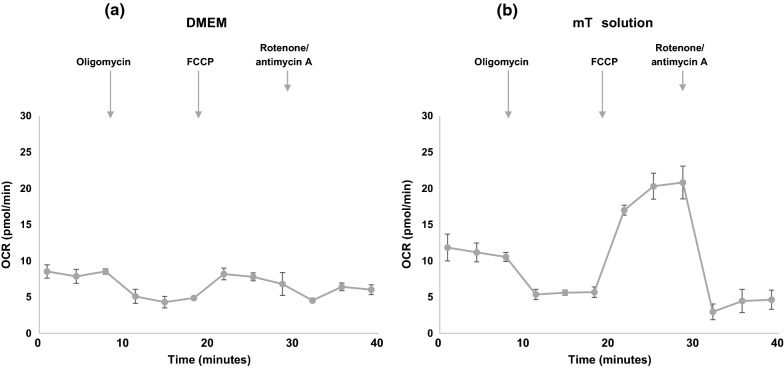
Optimization of the medium composition by assessing the OCR of human sperm mitochondria. The extracellular flux analysis was performed with sperm cells plated in (**a**) DMEM-based media or (**b**) mT solution

For measurement of oxygen consumption by human spermatozoa, the assay plates were treated with the candidate coating materials (i.e., concanavalin A, poly-D-lysine, and Cell-Tak adhesive) to immobilize sperm cells on the bottom well. Concanavalin A was selected as the primary option for sperm immobilization as it provided enhanced cell attachment as compared with the other commonly used coating reagents (Additional file 4: Figure S3). Next, we optimized the composition of assay media suitable for measuring mitochondrial respiration in human sperm. The XF Cell Mito Stress Test was performed with sperm cells plated either in DMEM-based media or mT solution. The changes in OCR in response to the modulators of cellular respiration were minimal when assayed in XF DMEM (Fig. 1a). Replacing the media with the mT solution significantly improved the results with a greater dynamic range for OCR values (Fig. 1b). Based on these observations, the mT solution was selected as a preferred medium for extracellular flux analysis using human sperm rather than the general DMEM.

### Optimization of the FCCP concentration

In the XF Mito Stress Test, the addition of FCCP, a potent uncoupler of mitochondrial oxidative phosphorylation, allows an estimate of the maximal respiratory capacity of mitochondria. Three doses (i.e., 0.5, 1.0 and 2.0 µM) of FCCP were tested to determine optimal concentration that can achieve maximal stimulation of OCR. The injection of FCCP at a dose of 0.5 µM yielded the highest OCR, and the value decreased at higher concentrations of FCCP (Additional file 5: Figure S4). Thus, 0.5 µM was chosen as the optimal concentration of FCCP for human sperm and used for further analysis.

### The impact of storage conditions on sperm motility and mitochondrial respiration

**Fig. 2:**
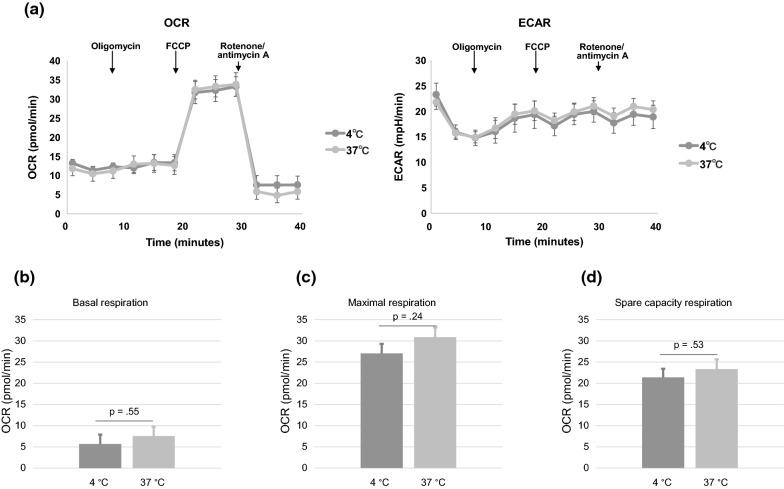
Metabolic analysis of human sperm. The motile human sperm OCR under different conditions (i.e., incubated at 4 or 37 °C for 2 h) was analyzed by the Extracellular Flux Analyzer™. **a** The XF Cell Mito Stress Test to monitor changes in OCR and ECAR in response to the metabolic modulators. **b** Basal respiration. **c** Maximal respiration. **d** Spare respiratory capacity

Two storage conditions were examined following isolation and collection of motile sperm: (i) incubation at 4 °C for 2 h and (ii) incubation at 37 °C for 2 h. Although no significant difference in sperm vitality was observed under both conditions (81.4% vs. 86.5%; *p* = 0.14), sperm motility of sperm incubated at 4 °C was lower than sperm incubated in 37 °C (61.5% vs. 82.5%; *p* = 0.031) (Table 1). The OCR under both conditions clearly increased upon addition of 0.5 µM FCCP with a maximum respiration rate of > 30 pmol/min reached. The OCR decreased after the addition of antimycin A/rotenone, indicating that respiration capacity was not exhausted during the storage (Fig. 2a). No significant difference in basal respiration, maximal respiration or spare respiratory capacity of human sperm under the two conditions was observed (Fig. 2b–d). The basal ECAR values, indicative of glycolytic activity, were comparable between the two storage conditions (Fig. 2a).

**Table 1:** Characteristics of semen samples

Original semen sample			
Semen volume, mL		2.8	
Sperm cell concentration, × 10^6^		81.3	
Total sperm motility, %		68.6	
Relative abundance, %	WHO A, B	62.2	
	WHO C	6.34	
	WHO D	31.4	
		Temperature	
After 2 h of incubation		4 °C	37 °C
Sperm vitality, %		81.4	86.5
Sperm motility, %		62.8	81.2

Data represent the mean values of three samples before and after 2 h of incubation at the indicated temperature

## Discussion

This study established extracellular flux analysis of oxygen consumption by human spermatozoa using the Extracellular Flux Analyzer™. Using this novel sperm quality assessment method, we showed that sperm motility of semen stored at low temperature (4 °C) for 2 h was significantly lower when compared with sperm motility of semen stored at a higher temperature (37 °C). In contrast, no difference in the OCR was observed. These results showed that mitochondrial respiration by highly active, normal human spermatozoa was maintained under both storage conditions. In addition, no difference in sperm vitality was observed under both conditions.

Sperm mitochondrial activity plays an important role in ensuring normal sperm function and energy homeostasis by oxidative phosphorylation and ATP synthase [2], and sperm motility is directly dependent on available energy provided by ATP hydrolysis [2]. Ruiz-Pesini et al. showed that the mitochondrial membrane potential and OCR are positively associated with ATP content, the proportion of motile sperm and sperm velocity [2]. Therefore, determining the relationship between sperm mitochondrial function and sperm quality is important for assessing the quality of human sperm. Sperm with higher motility have a higher fertilization rate during micro-insemination [10], whereas mitochondrial DNA deletion is associated with reduced motility and DNA fragmentation of sperm [11, 12].

Previously, Tourmente et al. showed that mice sperm produced more ATP and swam faster when they had a high respiration/glycolysis ratio and a high reliance on respiration. This previous study also showed that the usage ratio of ATP production pathways defines sperm motility in mice, and revealed the utility of extracellular flux analysis for examining oxygen consumption and extracellular acidification of spermatozoa [3]. In this study, we focused on using extracellular flux analysis to validate those previously reported observations in humans. Thus, our report is a seminal study examining sperm quality in relation to mitochondrial metabolism.

Extracellular flux analysis of human spermatozoa showed that there was no significant difference in the OCR at the two temperatures examined. Thus, although a decrease in sperm motility was observed at low temperature (4 °C), the sperm mitochondrial OCR was maintained over the temperature range (4–37 °C) and storage period (≤ 2 h) examined. These results indicated that mitochondrial ATP synthase activity is maintained at low temperatures for a minimum of 2 h. Sperm spare respiratory capacity was approximately three times higher than basal respiration (Fig. 2d), which revealed that there is considerable spare capacity in human sperm oxygen consumption.

The WHO laboratory manual for the examination and processing of human semen recommends the following for semen sample collection: (i) the sample should be collected after a minimum of 2 days and a maximum of 7 days of sexual abstinence; (ii) the man should deliver the sample to the laboratory within 1 h of collection, and (iii) the specimen container should be kept at ambient temperature, i.e., between 20 and 37 °C [1]. These recommendations should ensure sperm quality. This recommendation is acceptable based on our assessment of the sperm mitochondrial OCR.

Our study indicate that the quality of the sperm should not be assessed immediately after taking delivery of the semen sample from the patient but after incubating the sample at 37 °C for at least 30 min to ensure an accurate reading of normal mitochondrial function. In assessing human sperm quality of infertile couples, the approach to prepare sperm samples used in this study is recommended.

## Conclusion

This study established a human sperm quality evaluation technology using oxygen metabolism in mitochondria and showed that the OCR was maintained over the temperature range of 4–37 °C for a duration of 2 h. In addition to conventional functional indicators such as motility and survival rate, this novel technology can be potentially developed into a novel quantitative and objective evaluation method of sperm quality.

## Limitations

The limitations of this study include a small number of semen samples, analysis of a single participant with only normal sperm quality according to the WHO 2021 criteria and the examination of only motile sperm. Despite these limitations, we have established a novel human sperm quality evaluation technology using oxygen metabolism in mitochondria of human sperm.

## Supplementary Information

**Additional file 1: Table S1.** Key resources.

**Additional file 2: Figure S1.** XF cell mito stress test.

**Additional file 3: Figure S2.** Flow chart of the extracellular flux analysis of human spermatozoa.

**Additional file 4: Figure S3.** Representative images of sperm cells seeded on uncoated or concanavalin A-coated XF plates.

**Additional file 5: Figure S4.** Optimization of the FCCP reagent concentration.

## Data Availability

The datasets generated and/or analyzed during the current study are not publicly available due to relevant data protection laws but may be available from the corresponding author on reasonable request.

## Abbreviations

ATP: Adenosine triphosphate
DMEM: Dulbecco’s modified eagle medium
DNA: Deoxyribonucleic acid
FCCP: Carbonyl cyanide-p-trifluoromethoxyphenylhydrazone
IVF: In vitro fertilization
mT: Modified Tyrode’s
OCR: Oxygen consumption rate
SMAS: Sperm Motility Analysis System
WHO: World Health Organization
