# Supplementary material for: Establishment of a novel assessment of the quality of human spermatozoa measuring mitochondrial oxygen metabolism
Source: BMC Res Notes. 2022 Mar 29;15:123. doi: 10.1186/s13104-022-06012-4 (PMC8966288; doi:10.1186/s13104-022-06012-4)
Supplement: Supplementary file 1 — Additional file 1: Table S1. Key resources. [file 13104_2022_6012_MOESM1_ESM.pdf]

Reagents	Source	Identifier
antimycin A	Sigma-Aldrich	A8674
carbonyl cyanide 4-(trifluoromethoxy) phenylhydrazone (FCCP)	Sigma-Aldrich	C2920
concanavalin A	Sigma-Aldrich	C2010
oligomycin	Sigma-Aldrich	O4876
rotenone	Sigma-Aldrich	R8875
XF Dulbecco's Modified Eagle Medium (DMEM) medium	Agilent	103575-100
Isolate stock solution	Irvine Scientific	99275
Universal IVF Medium	Origio	10311010A

Supplementary Table 1 Key Resources
