# Supplementary material for: Establishment of a novel assessment of the quality of human spermatozoa measuring mitochondrial oxygen metabolism
Source: BMC Res Notes. 2022 Mar 29;15:123. doi: 10.1186/s13104-022-06012-4 (PMC8966288; doi:10.1186/s13104-022-06012-4)
Supplement: Supplementary file 2 — Additional file 2: Figure S1. XF cell mito stress test. [file 13104_2022_6012_MOESM2_ESM.pdf]

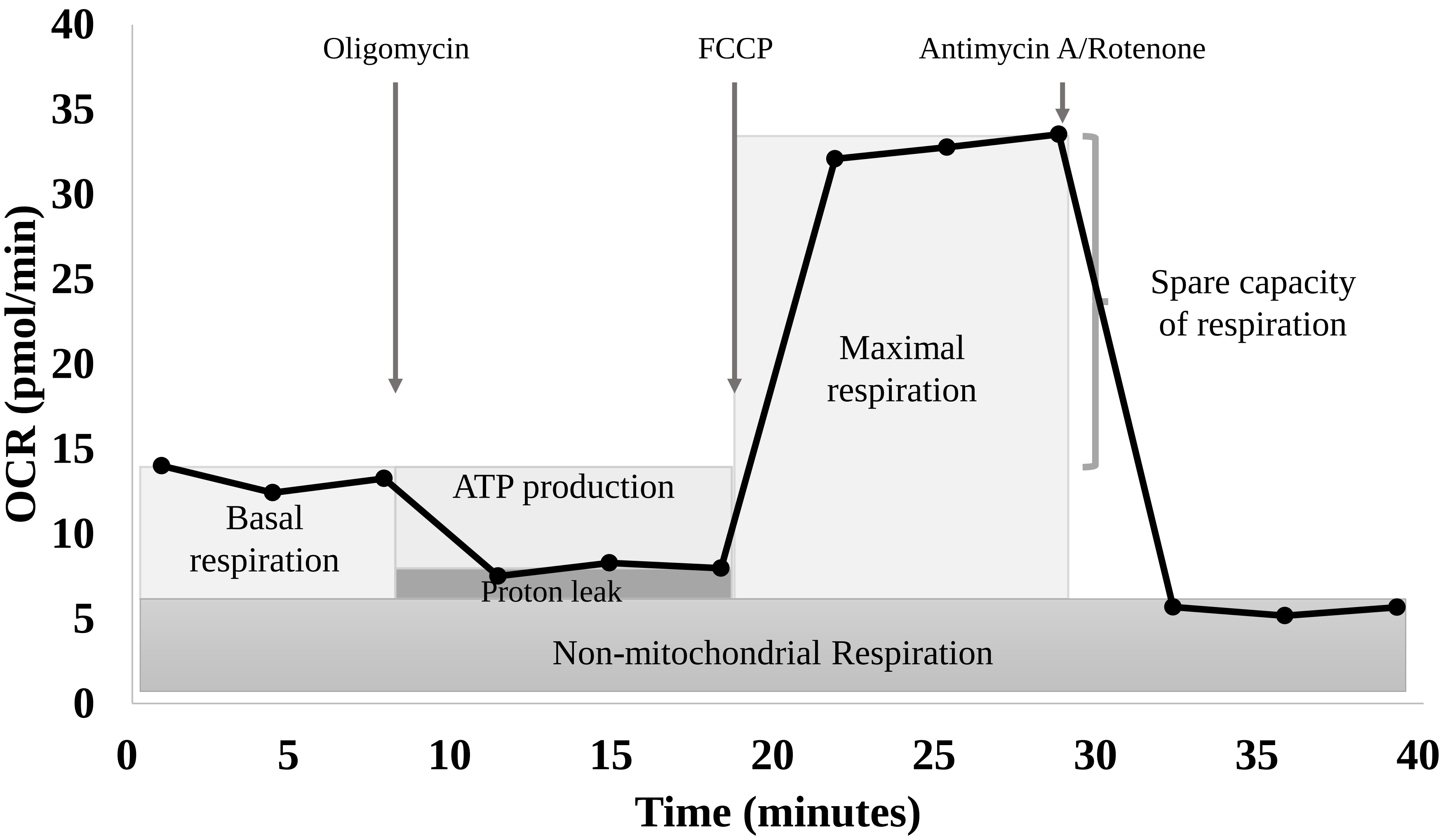

Supplementary Figure 1: XF Cell Mito Stress Test.

XF Cell Mito Stress Test profile showing the key features of the mitochondrial OCR
