# Supplementary figures and images for: Establishment of a novel assessment of the quality of human spermatozoa measuring mitochondrial oxygen metabolism
Source: BMC Res Notes. 2022 Mar 29;15:123. doi: 10.1186/s13104-022-06012-4 (PMC8966288; doi:10.1186/s13104-022-06012-4)

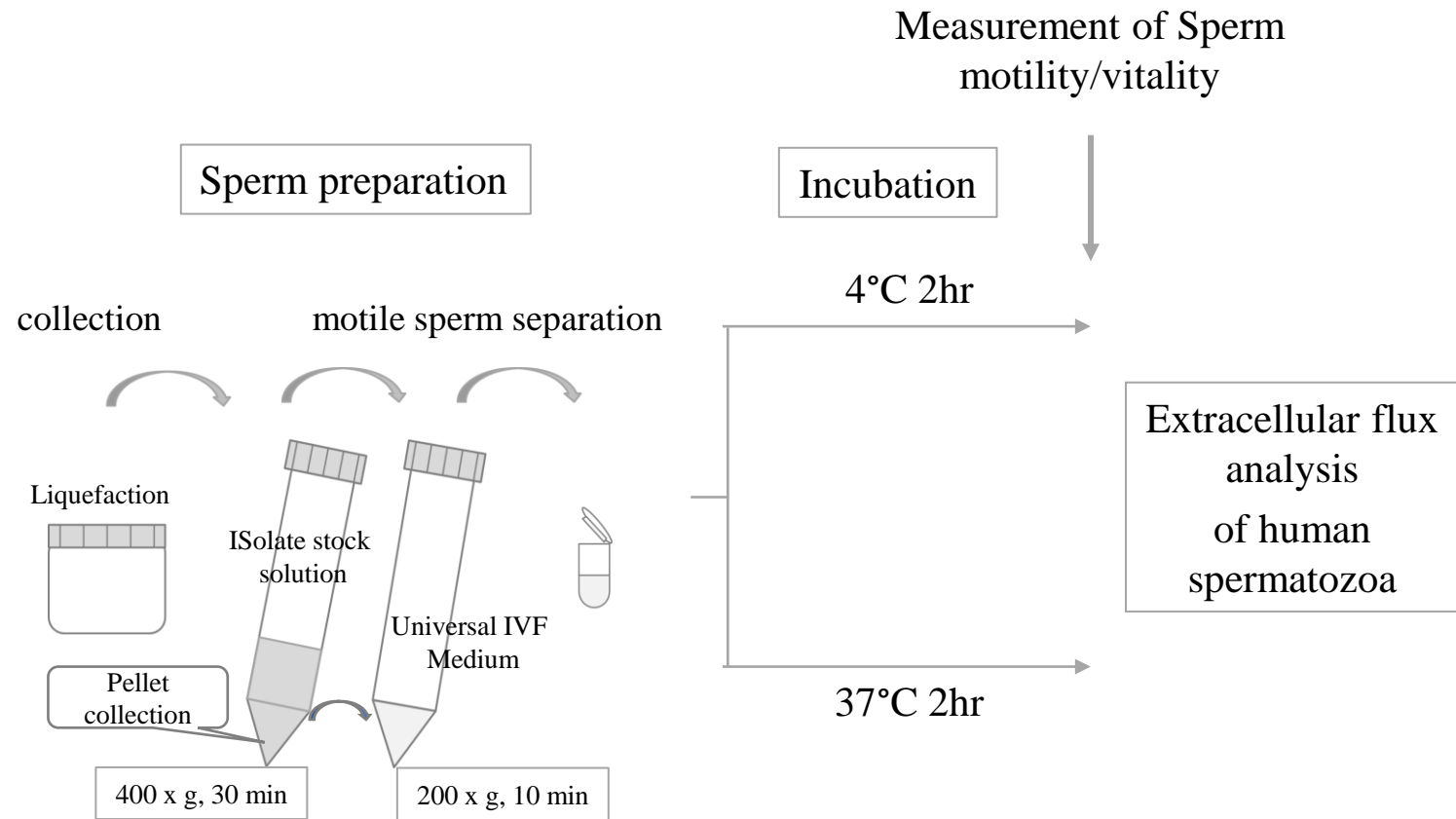

**Supplementary Figure 2: Flow chart of the extracellular flux analysis of human spermatozoa.**

Supplement: Supplementary file 3 — Additional file 3: Figure S2. Flow chart of the extracellular flux analysis of human spermatozoa. [file 13104_2022_6012_MOESM3_ESM.pdf]
