# Supplementary material for: Establishment of a novel assessment of the quality of human spermatozoa measuring mitochondrial oxygen metabolism
Source: BMC Res Notes. 2022 Mar 29;15:123. doi: 10.1186/s13104-022-06012-4 (PMC8966288; doi:10.1186/s13104-022-06012-4)
Supplement: Supplementary file 4 — Additional file 4: Figure S3. Representative images of sperm cells seeded on uncoated or concanavalin A-coated XF plates. [file 13104_2022_6012_MOESM4_ESM.pdf]

(a) Uncoated (b) Concanavalin A

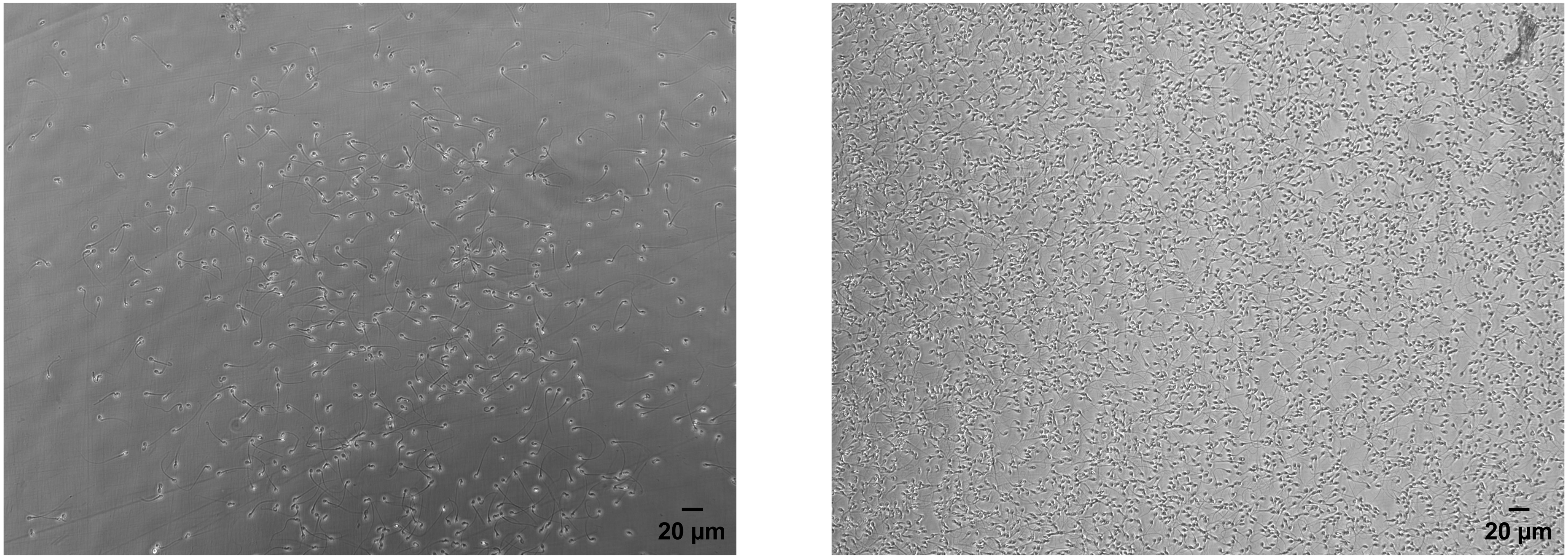

Supplementary Figure 3. Representative images of sperm cells seeded on (a) uncoated or (b) concanavalin A-coated XF plates.
