# Supplementary material for: Establishment of a novel assessment of the quality of human spermatozoa measuring mitochondrial oxygen metabolism
Source: BMC Res Notes. 2022 Mar 29;15:123. doi: 10.1186/s13104-022-06012-4 (PMC8966288; doi:10.1186/s13104-022-06012-4)
Supplement: Supplementary file 5 — Additional file 5: Figure S4. Optimization of the FCCP reagent concentration. [file 13104_2022_6012_MOESM5_ESM.pdf]

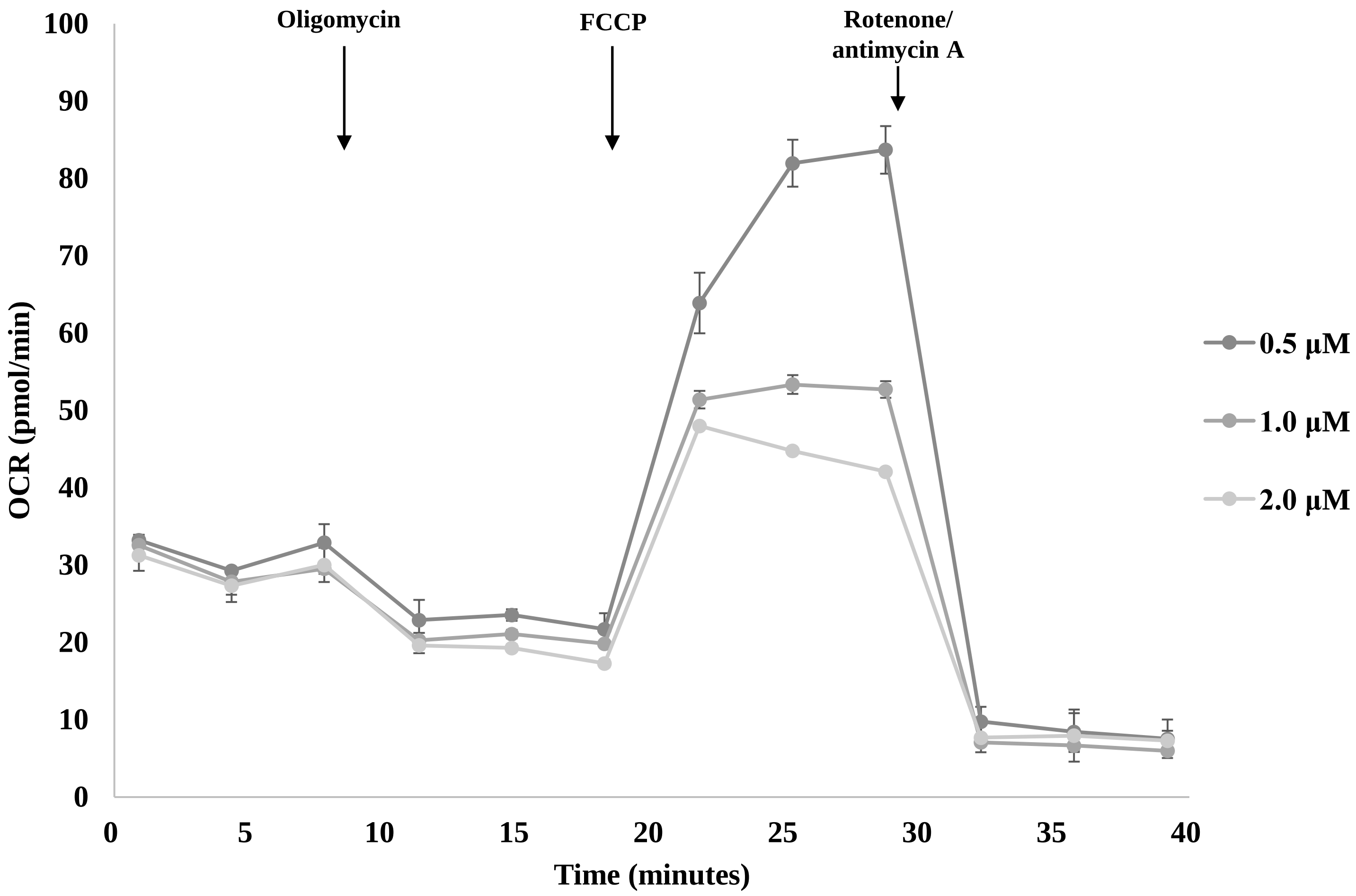

Supplementary Figure 4: Optimization of the FCCP reagent concentration.

FCCP was titrated (0.5, 1.0, 2.0 µM) to achieve maximal stimulation of OCR.
